# Biological and clinical implications of cancer stem cells in primary brain tumors

**DOI:** 10.3389/fonc.2013.00006

**Published:** 2013-01-25

**Authors:** Marcello Maugeri-Saccà, Simona Di Martino, Ruggero De Maria

**Affiliations:** “Regina Elena” National Cancer InstituteRome, Italy

**Keywords:** glioblastoma multiforme, cancer stem cells, chemo-radioresistance, canonical pathway inhibitors, self-renewal pathway inhibitors, differentiation-inducing agents, hypoxia, stem cell-based endpoints

## Abstract

Despite therapeutic advances, glioblastoma multiforme (GBM) remains a lethal disease. The infiltrative nature of this disease and the presence of a cellular population resistant to current medical treatments account for the poor prognosis of these patients. Growing evidence indicates the existence of a fraction of cancer cells sharing the functional properties of adult stem cells, including self-renewal and a greater ability to escape chemo-radiotherapy-induced death stimuli. Therefore, these cells are commonly defined as cancer stem cells (GBM-SCs). The initial GBM-SC concept has been challenged, and refined according to the emerging molecular taxonomy of GBM. This allowed to postulate the existence of multiple CSC types, each one driving a given molecular entity. Furthermore, it is becoming increasingly clear that GBM-SCs thrive through a dynamic and bidirectional interaction with the surrounding microenvironment. In this article, we discuss recent advances in GBM-SC biology, mechanisms through which these cells adapt to hostile conditions, pharmacological strategies for selectively killing GBM-SCs, and how novel CSC-associated endpoints have been investigated in the clinical setting.

## INTRODUCTION

Glioblastoma multiforme (GBM, World Health Organization grade IV gliomas) is the most common and lethal form of primary brain tumors. Despite multimodality management, consisting in surgery followed by adjuvant chemo-radiotherapy with temozolomide, GBM remains a devastating disease with a 2-year survival rate in the range of 10–25% (Stupp et al., 2005). Although the use of molecular targeted agents in defined genetic backgrounds is changing the treatment of many solid tumors, GBM continues to be an orphan disease. The impact on the overall survival (OS) rate achieved with the anti-vascular endothelial growth factor (VEGF) monoclonal antibody bevacizumab, the only molecular targeted agent currently approved by the FDA, has not yet been understood (reviewed in Patel et al., 2012). Furthermore, many small molecule inhibitors stopped the clinical developmental path prematurely, despite an apparently solid biological background underlying their evaluation in GBM, whereas immunotherapies and toxin-ligand conjugates, which have shown promise in early clinical trials, require larger randomized studies. Apart from these disappointing results, major breakthroughs in recent years have significantly contributed in deciphering the biology of GBM. Firstly, the molecular taxonomy of the disease is rapidly expanding. The Cancer Genome Atlas Network recently cataloged genomic defects of GBM. These efforts allowed to classify GBM into distinct molecular entities, each one characterized by a specific gene expression profile and a different set of mutant genes (Verhaak et al., 2010). The identification of proneural, neural, classical, and mesenchymal subtypes suggested that each subtype should be approached as a distinct disease, given the potential diversity in the disease’s course and, even more importantly, in its sensitivity to pathway-focused inhibitors. As a result, novel molecular classifiers are expected to turn GBM into multiple rare diseases, thus revolutionizing the current clinical trial methodology that will require novel endpoints and enrichment strategies. Secondly, our knowledge on the biological mechanisms underlying tumor formation and evolution have been implemented with the discovery of a cellular hierarchy within the tumor, which makes only a fraction of cancer cells worth targeting. The cellular pool responsible for tumor propagation possesses stem-like traits, consequently these cells have been defined as cancer stem cells (CSCs). CSCs have been successfully isolated in most hematological malignancies and solid tumors, including GBM (Bonnet and Dick, 1997; Al-Hajj et al., 2003; Singh et al., 2003; Ricci-Vitiani et al., 2007; Eramo et al., 2008; Todaro et al., 2010). In this article, we discuss the origin and evolution of the CSC concept applied to primary brain tumors, pharmacological strategies endowed with potential anti-CSC properties, and how the CSC concept has been applied to the clinic.

## THE EVOLUTION OF THE CANCER STEM CELL MODEL

**FIGURE 1:**
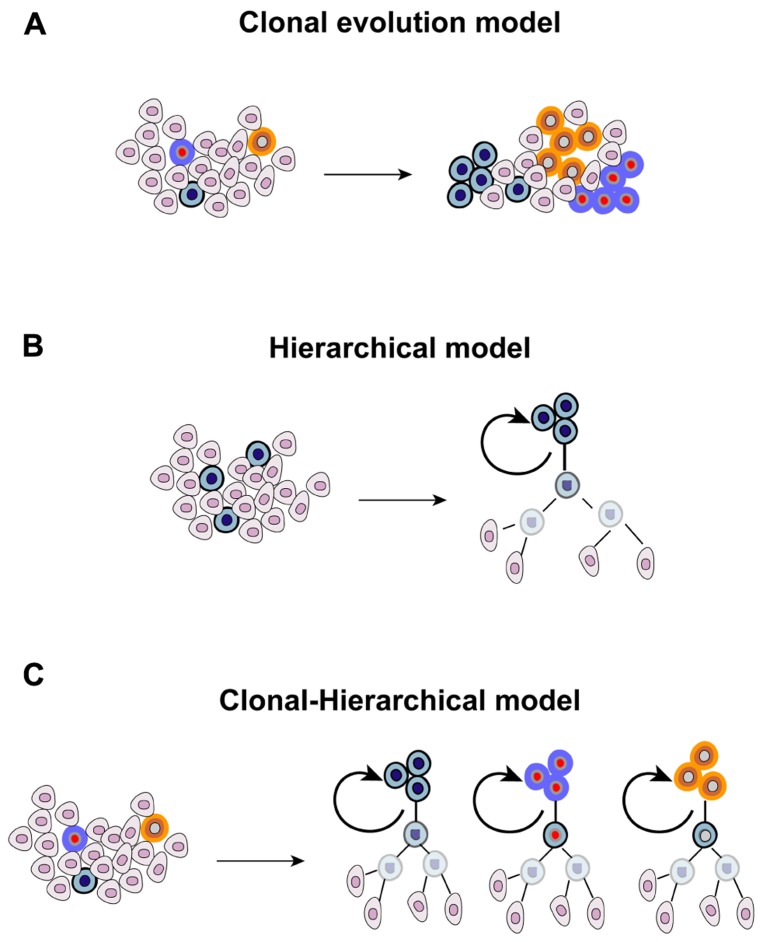
**Theories proposed for explaining the origin and evolution of cancer**.**(A)** Different mutant clones cohabit the tumor, each one with the same ability to proliferate and to retain tumorigenicity, and the random occurrence of genetic events confers dominant traits to some of them, **(B)** the CSC model originally postulated that a stem-like cell located at the apex of the tumor pyramid is the precursor of the whole tumor population, and **(C)** the combined clonal-stem cell model suggests that CSCs can undergo clonal evolution, and therefore multiple CSC clones coexist within the tumor.

The heterogenic nature of tumors is evident in the clinical setting, where different lesions present different, or even opposite, patterns of response following systemic anticancer therapy. This has been recently documented at the molecular level. Different metastatic sites possess, other than a common panel of mutations, also ”private“ alterations that make them unique (Gerlinger et al., 2012). Tumor heterogeneity has emerged since the earliest pathological examinations, and originally explained in 1858 by Rudolf Virchow who proposed that tumors arise from embryo-like cells (Virchow, 1855). This theory was further refined by Cohnheim and Durante with the ”embryonal rest theory“ (Durante, 1874; Cohnheim, 1875). According to this model, embryonic remnants are present throughout the body, usually lying in a dormant state but, when reactivated, they give rise to tumors. The heterogenic nature of cancer and the dynamics existing within a tumor cell population have been connected to evolutionary principles and shaped upon Darwinian laws. Each clone has the same ability to proliferate and to retain tumorigenicity. However, the random occurrence of mutations leads to the emersion of dominant clones that acquire a survival advantage over other cells, having a greater ability to thrive in a hostile microenvironment and to adapt to microenvironmental perturbations, ultimately prevailing over cells that do not acquire these advantageous traits (”stochastic clonal evolution model“). In recent years, a novel and seemingly contradictory model was proposed following the identification and characterization of a rare fraction of tumorigenic cancer cells resembling normal stem cells, thanks to their ability to self-renew and to differentiate into different lineages. The CSC model postulated the existence of a rigid and immutable hierarchy within a tumor (”hierarchical model“), which is organized in a pyramidal manner with few CSCs at the apex representing the founders of the entire population. However, the antithetic nature of the ”clonal evolution“ and ”hierarchical“ models was later excluded with an in-depth characterization of embryonic, adult, and CSCs. The fact that the ”stemness“ state can be induced in differentiated cells was originally demonstrated with the forced expression of four embryonic stem cell-specific transcription factors in fibroblasts (Takahashi and Yamanaka, 2006). Subsequently, also the CSC state appeared to fluctuate, and be affected by multiple conditions including hypoxia, low pH, exposure to paracrine-acting signals such as stimulation with hepatocyte growth factor, and activation of the epithelial-mesenchymal transition (EMT) program (Mani et al., 2008; Li et al., 2009; Vermeulen et al., 2010; Hjelmeland et al., 2011). Under these conditions, differentiated cancer cells acquire the operative criteria of CSCs, including the expression of stem cell markers and clonogenic ability determined with limiting-dilution assay. It is therefore foreseeable that exogenous influences are involved in the process of maintaining and enriching CSCs, highlighting the fact that the retention/acquisition of stem-like features is a dynamic process. Furthermore, microenvironmental influences, which represent a mainstay of evolutionary principles, also affect the biology of CSCs. Also the original rigidity of the CSC model that stated that only stem-like cells can propagate the tumor was reviewed. Not only CSCs, but also their proximal progeny, can propagate the tumor although with a different temporal pattern (Dieter et al., 2011). Finally, the genetic heterogeneity of cancer propagating cells suggests a clonal evolution within the stem cell pool (Anderson et al., 2011). Overall, evidence mentioned above allowed to envision a combined clonal-stem cell model (**Figure 1**). The first hint supporting the existence of CSCs in GBM was provided in 2003 and 2004 (Singh et al., 2003, 2004), and confirmed by independent research groups (Galli et al., 2004). These studies revealed the existence of a rare fraction of cancer cells able to self-renew, as measured by neurosphere-formation assay and tumor propagation *in vivo* in intracranial limiting dilution assays. The first wave of investigation conducted with GBM stem cells (GBM-SCs) suggested that only the CD133^+^ population was endowed with clonogenic ability and was able to recapitulate the parental disease after inoculation in immunocompromised mice. However, subsequent studies revealed that the stem-like state is not restricted to CD133^+^ cells, but also CD133^-^ cells can fulfill criteria to be defined as CSCs (Beier et al., 2007; Piccirillo et al., 2009), a finding that is consistent with studies performed with other CSC types (Shmelkov et al., 2008). These observations suggested that CD133 cannot be defined as a universal marker for GBM-SCs, and raised the hypothesis that it might serve for defining a subgroup of brain tumor stem cells, potentially identifying a given molecular entity. Furthermore, the presence of both CD133^+^ and CD133^-^ self-renewing tumor-initiating cells within a tumor indicates the coexistence of multiple CSC clones, a finding substantiated by differences in gene expression and growth kinetics of orthotopic grafts between CD133^+^ and CD133^-^ cells (Chen et al., 2010). The cytoarchitecture of GBM, consisting in normoxic cells in the periphery, hypoxic cells in the center and necrotic cells in the inner core, further suggested that different microenvironmental conditions might affect CSC properties. Consistent with this, cells residing in the inner core and in the intermediate layer display a more immature phenotype, and possess greater clonogenic ability compared with more peripheral cells (Pistollato et al., 2010). However, the interaction of GBM-SCs with the surrounding non-cancerous tissue is bidirectional. If, on the one hand, microenvironment factors influence the biological behavior of GBM-SCs, on the other hand these cells demonstrated the ability to recreate more favorable conditions, as demonstrated by their ability to actively participate in the generation of new blood vessels through producing angiocytokines, and their direct differentiation into endothelial-like cells (Bao et al., 2006a; Ricci-Vitiani et al., 2010; Wang et al., 2010). Overall, the diversity existing in the CSC pool highlights the increasing complexity of the CSC paradigm in GBM, and the importance of gaining a deeper understanding of the evolutionary dynamics at the apex of the tumor pyramid.

## CONTROVERSIES IN GLIOBLASTOMA MULTIFORME STEM CELLS

The ”stem cell-centric“ model of cancer originally envisioned an adult stem cell as the target of oncogenic hits. Accordingly, the malignant transformation of these cells gives rise to cancer cells that maintain stem-like traits. This has fostered the translation of knowledge about stem cell biology to the pathobiology of cancer, allowing the identification of CSC-restricted pathways valuable for pharmacological inhibition, and to define a fraction of cancer cells with increased resistance to chemo-radiotherapy compared to the bulk of tumor cell mass. Nevertheless, there are much controversies over the existence, origin, and nomenclature of CSCs (Lathia et al., 2011; Valent et al., 2012). The link existing between adult stem cells and CSCs has been the focus of many investigations. Although genetically engineered mouse models provided hints that GBM originates from the malignant transformation of neural stem/progenitor cells (reviewed in Lathia et al., 2011), these results should be interpreted with caution. For instance, current animal models did not exclude the possibility that also non-stem cells can give rise to GBM, when manipulated with multiple and sequential mutational hits. Consistent with this, recent studies demonstrating that GBM can be generated by cells that do not reside within neurogenic niches (Zhu et al., 2009). Functionally, CSCs are defined as a subpopulation of tumor-initiating cells having the ability to reconstitute the cellular heterogeneity typical of the original tumor. Operatively, to define CSCs some criteria need to be fulfilled, such as expression of a repertoire of markers common to stem and progenitor cells, ability to self-renew, and capability to reproduce the parental tumor upon injection into immunocompromised mice. Although informative, the expression of a single marker cannot be considered as a general principle for defining CSCs, nor a somatic stem cell. As discussed above, CD133 expression was originally used for GBM-SCs characterization. Nevertheless, this criterion nowadays appears to be oversimplified, while the validation of novel markers and their combined use might add a further level of reliability to current isolation protocols. The ability to self-renew and to differentiate into multiple lineages is a hallmark of stem cells, enabling them to maintain tissue homeostasis and to replace senescent or dying cells. Self-renewal of adult stem cells is determined through sphere-forming ability under non-adherent culture conditions. Given the analogies supposed to exist between adult stem cells and CSCs, the same assay is used for defining putative CSCs. Even though spheroids are enriched for CSCs, one should consider that tumorsphere-formation assay does not provide an exact, nor an exclusive, measurement of self-renewal ability, since this assay evaluates further biological properties, such as adhesion independence, survival, and proliferation. Overall, the ability to recreate the original tumor upon the delivery into the murine background represents the most critical factor in defining CSCs. Furthermore, our unpublished data suggests that CSCs generates tumors maintaining the original molecular portrait of the parental neoplasia, as demonstrated by mapping pathway activation through reverse-phase phosphoprotein microarray. Finally, microarray analyses demonstrated the intrinsic heterogeneous nature of GBM. This suggests that each molecular entity might stem from a different cell of origin (Phillips et al., 2006; Verhaak et al., 2010). Overall, further preclinical investigations are needed for resolving controversies surrounding the CSC model in GBM, and for acquiring a deeper understanding of subtype-restricted molecular signals.

## BRAIN TUMOR STEM CELLS AND RESISTANCE TO CURRENT ANTICANCER THERAPIES

Growing evidence indicates that CSCs are less vulnerable to chemo- and radiotherapy than the bulk of tumor cells (reviewed in Maugeri-Saccà et al., 2011a). Among multiple mechanisms that defend CSCs against harmful insults, is the capacity to rapidly correct genetic lesions generated by DNA damaging agents, such as ionizing radiation and alkylating agents (reviewed in Maugeri-Saccà et al., 2012). Radioresistance of GBM-SCs was originally connected with enhanced DNA repair ability. GBM-SCs showed a more effective activation of DNA damage checkpoints compared with tumor cells without stem cell properties, a phenomenon reverted with the abrogation of the cell cycle checkpoint controllers Chk1 and Chk2 (Bao et al., 2006b). However, this finding was later questioned. Although enhanced activation of Chk1 and Chk2 was found in untreated CD133^+^ compared to CD133^-^, the stem cell fraction did not display increased DNA repair proficiency (Ropolo et al., 2009). Evidence that the population doubling time was significantly increased in GBM-SCs compared to non-stem cells suggested that their radioresistance is due to an elongated cell cycle, resulting from enhanced basal activation of checkpoint proteins. The biological relevance of the DNA damage machinery was further extended by proving that the self-renewal-linked protein Bmi1 mediates radioresistance through the recruitment of multiple DNA damage repair components (Facchino et al., 2010). Consistently, Bmi1 deficiency increased sensitivity to radiation consequently to impaired DNA double-strand break response. This picture was further complicated by comparing radioresistance between GBM-SCs and established cell lines. Although within a subset of CSCs the CD133^+^ pool was more resistant to ionizing radiation as compared to CD133^-^, CSCs displayed impaired DNA repair and were more sensitive to irradiation than commercial cell lines (McCord et al., 2009). Beyond DNA repair capacity, additional mechanisms have been associated with radioresistance. Irradiation of glioma cells induces autophagy to a wider extent in the CD133^+^ compartment, which expresses higher levels of autophagy-related proteins (Lomonaco et al., 2009). Induction of autophagy conferred radioresistance to these cells, a property abolished with autophagy inhibitors that decreased survival and neurosphere-forming ability. The activation of self-renewal pathways, such as Notch and Wnt, has been associated with radioresistance (Wang et al., 2010; Kim et al., 2012). Notably, gamma-secretase and Wnt inhibitors sensitized GBM-SCs to radiation, decreasing clonogenic ability and impairing xenograft formation. Also extrinsic mechanisms play a role in the ability of GBM-SCs to survive ionizing radiation. Cells irradiated *in vivo* accumulate less genetic damages and express higher levels of genes related to reactive oxygen species metabolism as compared to *in vitro* irradiated cells (Jamal et al., 2010). This indicates that microenvironmental cues may affect the susceptibility to ionizing radiation by improving DNA repair capacity. The notion that current medical treatments preferentially kill non-CSCs allowed to postulate an enrichment of the stem cell compartment following exposure to radio- and chemotherapy. Even though explored in a small study cohort, accumulation of CD133^+^ cells was documented after stereotactic radiosurgery and external beam radiation therapy, thus suggesting that glioma stem-like cells can survive high-dose irradiation (Tamura et al., 2010). While radioresistance of GBM-SCs is widely accepted, the question whether or not CSCs account for the limited efficacy of chemotherapy in GBM still remains controversial. Despite findings from studies investigating the sensitivity of GBM-SCs to chemotherapy result inconclusive, with some reports describing an increased resistance (Eramo et al., 2006) and others an increased susceptibility (Beier et al., 2008), all studies reported an overall increased resistance to temozolomide of MGMT-expressing GBM-SCs (reviewed in Beier et al., 2011). Furthermore, temozolomide resistance has been associated with the intratumoral hypoxic gradient. While CSCs residing in the periphery showed susceptibility to temozolomide, CD133^+^ cells located in the inner core were resistant and displayed the highest expression of MGMT (Pistollato et al., 2010). More recently, investigators identified a restricted cell population capable to repopulate the tumor following chemotherapy using a genetically engineered mouse model of glioma, thus providing novel hints on the contribution of CSCs on tumor recurrence despite adjuvant therapy (Chen et al., 2012). Pharmacological abrogation of stem cell-restricted pathways seems to enhance the efficacy of standard chemotherapeutics. Inhibition of either Hedgehog or Notch pathway modulates the effects of temozolomide in CSCs potentiating its antitumor activity, thus setting the biological basis for testing these combinations in clinical trials (Gilbert et al., 2010; Ferruzzi et al., 2012). Additional molecular mechanisms have been associated with the ability of CSCs to survive standard chemotherapeutic agents. Among these are the increased function of ATP-binding cassette transporters, a system committed to actively transporting chemicals out of the cells, and a tendency toward an anti-apoptotic state (reviewed in Maugeri-Saccà et al., 2011b). Also, microenvironmental stimuli contribute to chemo-radioresistance. The EMT is an evolutionarily conserved process that is essential for tissue remodeling during morphogenesis (Lim and Thiery, 2012). The activation of EMT-associated transcription factors leads to a drastic cytoskeletal rearrangement, through which epithelial cells lose their polarized organization and cell–cell junctions. Therefore, cells undergoing EMT acquire a high motile, mesenchymal-like phenotype. Several lines of evidence connected EMT with cancer invasion, metastasis, ability to evade the host immune response to the tumor, resistance to current cancer therapeutics and CSC generation (reviewed in Tiwari et al., 2012). While EMT induction has been prominently associated with the paracrine-acting transforming growth factor β (TGF-β) pathway (Tiwari et al., 2012), a wave of preclinical investigations revealed that a similar outcome is elicited by the self-renewal-associated pathways Sonic Hedgehog (Shh), Notch, and Wnt (Ohta et al., 2009; Wang et al., 2009; Gupta et al., 2010). Recently, in squamous cell carcinoma investigators revealed the coexistence of CSCs with either epithelial or mesenchymal traits, and endowed with distinct biological properties (Biddle et al., 2011). This finding was in line with the ”migrating cancer stem cell“ concept (Brabletz et al., 2005 ), according to which the transient expression of EMT-related genes enable CSCs to migrate to a distant site, where the opposite path takes place (mesenchymal-epithelial transition). This epithelial re-differentiation leads to tumor growth in a foreign soil, and to the generation of lesions with the same epithelial characteristics as the original tumor. Although intriguing, it is worth considering that controversies exists around the role of EMT (reviewed in Bastid, 2012). These fundamentally stem from the difficulty of providing direct evidence of EMT in human tumor specimens, which cannot be fully explained by the presence of sarcomatoid elements in epithelial cancers.

## TARGETING GLIOBLASTOMA MULTIFORME STEM CELLS

Refining protocols for CSCs isolation and expansion has enabled investigators to identify CSC-restricted molecular networks valuable for pharmacological abrogation. Schematically, compounds with potential anti-GBM-SCs activity can be classified as molecules targeting canonical and self-renewal pathways, agents inducing differentiation, and molecules depriving CSCs of microenvironmental stimuli.

## TARGETING CANONICAL PATHWAYS

The epidermal growth factor receptor (EGFR) signaling is one of the most common deregulated pathways in cancer. The mutant and constitutively active variant EGFRvIII, which is found in 25% of GBM and leads to increased Akt signaling (Choe et al., 2003), has been linked to GBM-SCs function. EGFRvIII seems to control GBM-SCs fate at multiple levels and through different molecular mechanisms. The EGFR-Akt-Smad5 axis induces the inhibitor of differentiation 3 (Id3) that, in turn, promotes neurosphere formation (Jin et al., 2011). Furthermore, a telomerase activity-deficient form of telomerase reverse transcriptase (TERT) was shown to induce EGFR expression in glioma cells, in a process coupled with the acquisition of stem-like traits (Beck et al., 2011). Consistent with the biological relevance of aberrant EGFR activation in glioma, GBM-SCs have been reported to be more dependent on Akt signaling than the non-CSC counterpart. This was demonstrated by the ability of pharmacological Akt inhibitors to disrupt neurosphere generation and delay intracranial tumor formation (Eyler et al., 2008). Even though acting downstream the pathway has been proposed as an effective way for GBM-SCs targeting (Bleau et al., 2009), it is worth considering that the phosphoinositide 3-kinase (PI3K)/AKT pathway inhibitor enzastaurin failed to demonstrate superiority over lomustine in a phase III trial conducted in patients with recurrent GBM (Wick et al., 2010). Moreover, further reports shed doubt on the therapeutic relevance of EGFR inhibition as anti-CSC strategies. For instance, EGFR abrogation failed to prevent tumorigenesis of transformed neural stem cells or GBM-SCs *in vivo* (Hide et al., 2011) and, more recently, EGFR inhibition in the mutant background paradoxically increased GBM-SCs malignancy (Jin et al., 2012). In addition, invasiveness of GBM-SCs did not correlate with PI3K-Akt pathway activation (Wakimoto et al., 2012). Recently, a compensatory activation of others ERBB family receptors was observed in GBM-SCs following EGFR abrogation, thus raising the possibility that efficient anti-EGFR therapy requires the co-targeting of multiple family members (Clark et al., 2012). To sum up, a deeper understanding of EGFR biology in CSCs, which considers the molecular taxonomy of the disease, is required for improving the anti-CSC properties of these agents. The TGF-β pathway is involved in multiple biological activities spanning from immunosuppression to migration and invasion of cancer cells. The ability of TGF-β and leukemia inhibitory factor (LIF) in preventing differentiation and in inducing self-renewal of GBM-SCs, but not of normal human neuroprogenitors, has been recently demonstrated via a molecular cascade requiring the activation of the JAK-STAT-pathway (Peñuelas et al., 2009). Consistently, the pro-CSC effects of the pathway were neutralized through TGF-β receptor I inhibition (Anido et al., 2010). Furthermore, a TGF-β-Sox4-Sox2 pathway was described to be essential in retaining the stemness state (Ikushima et al., 2009). In more detail, the stem cell marker Sox2 is induced by TGF-β via Sox4 activation, while pathway inhibition promotes differentiation, impairs tumorigenicity, and extends the survival of mice bearing CSC-generated orthotopic glioma. Interestingly, differences in the transcriptional activity of the TGF-β/bone morphogenetic protein signaling seem to identify distinct CSCs that give rise to different molecular subtypes of GBM (Lottaz et al., 2010). As mentioned above, STAT3 was identified as a regulator of self-renewal in GBM-SCs. STAT3 is a transcriptional regulator involved in a wide range of cellular activities such as immune response, stem cell maintenance, and tumorigenesis, and its association with gliomagenesis has become increasingly evident. STAT3 inhibition impairs GBM-SCs proliferation and disrupts stem cell maintenance (Sherry et al., 2009). Furthermore, the constitutively active STAT3 pathway in GBM-SCs contributes to the inhibition of T cell proliferation and activation, while this immunosuppressive status was diminished with STAT3 blockade (Wei et al., 2010). The list of potential pathway-focused inhibitors targeting deregulated canonical signals in GBM-SCs includes also MET inhibitors. The MET tyrosine kinase receptor is an established oncogenic signal known to stimulate survival, proliferation, and invasion of GBM cells. High levels of MET were found in GBM specimens topographically localized in perivascular regions. This fraction of MET-expressing cells was endowed with high clonogenic and tumorigenic ability, and displayed resistance to radiation, while MET inhibition hampered their growth and invasiveness both *in vitro* and *in vivo* (Joo et al., 2012). c-MET activation was also able to counteract the effects of forced differentiation (Li et al., 2011). Notably, MET expression was associated with neurospheres expressing the gene signature of mesenchymal and proneural subtypes, while it was absent in neurospheres expressing the classical subtype signature, being therefore mutually exclusive with EGFR abnormalities (De Bacco et al., 2012).

## TARGETING SELF-RENEWAL PATHWAYS

One of the assumption of the CSC model is that these cells share many functional properties with their normal counterpart. The Notch and Hedgehog pathways are essential for maintaining several types of adult stem cells, including neural stem cells (reviewed in Lathia et al., 2008; and in Ruiz iAltaba et al., 2007). Aberrant Notch signaling is common in tumors, and deregulated Notch activity has been linked to GBM-SCs. Notch inhibition achieved with gamma-secretase inhibitors (GSIs) hampered the formation of neurospheres *in vitro* and tumorigenicity *in vivo* (Fan et al., 2010). The depletion of CD133^+^ was accompanied by the reduction of putative CSC markers, and connected with decreased phosphorylation of AKT and STAT3. GBM-SCs are thought to reside within dedicated microarchitectonic entities that provide structural and functional support, commonly defined as vascular niches (Calabrese et al., 2007). By taking advantage of a three-dimensional model that preserves the cytoarchitecture of glioma and a functional stromal compartment, investigators revealed that endothelial cells modulate the self-renewal of GBM-SCs in a process disrupted by Notch inhibition (Hovinga et al., 2010). How Notch links angiogenesis and self-renewal is mechanistically explained by co-culturing Notch ligand-expressing human brain microvascular endothelial cells with neurospheres. Under these conditions endothelial cells promote growth and self-renewal of GBM-SCs, while the knockdown of Notch ligands abrogates this process (Zhu et al., 2011). Even though blocking Notch signaling with GSIs appears to be a valuable strategy, from which promising results have emerged from early clinical trials, it is worth considering that different Notch paralogs are known to possess different, or even opposite, biological functions. Since this notion potentially translates into either unnecessary side effects or reduced antitumor activity, more focused strategies for pathway inhibition have been explored. Nevertheless, the therapeutic potential of anti-Delta-like 4 therapies was counterbalanced by severe toxicity consisting in liver alterations and vascular neoplasms (Li et al., 2010; Yan et al., 2010). The Shh is a key regulatory pathway during embryogenesis. Recently, the first-in-class Smoothened inhibitor Vismodegib (GDC-0449) has been approved for treating basal-cell carcinoma, and displayed encouraging activity against medulloblastoma (Rudin et al., 2009; [Bibr B100]). The pathway is deregulated in multiple tumor types, and its aberrant activation sustains self-renewal and tumorigenic potential of GBM-SCs (Clement et al., 2007). Furthermore, antagonization of Shh pathway synergizes with temozolomide, leading to a more pronounced inhibition of GBM-SCs proliferation. A selective depletion of GBM-SCs was achieved with cyclopamine, as it has been further confirmed by reduced aldehyde dehydrogenase activity and Hoechst dye excretion (Bar et al., 2007). More importantly, viable cells injected intracranially following Shh inhibition failed to form a tumor, thus indicating an efficient targeting of tumor-propagating cells. In addition, the association of cyclopamine with the EGFR tyrosine kinase inhibitor erlotinib demonstrated synergistic activity against GBM-SCs (Eimer et al., 2012). Overall, these studies indicate that Shh pathway is crucial for regulating GBM-SCs fate, and that its abrogation may selectively target GBM-SCs, or render them more sensitive to current therapies.

## DIFFERENTIATION-INDUCING AGENTS

Induction of differentiation with retinoic acid is a therapeutic approach commonly used for treating hematological malignancies that has been also proposed in solid tumors. When exposed to retinoic acid GBM-SCs underwent both growth arrest and expression of lineage-specific differentiation markers, in a process associated with the down-regulation of Notch-related genes (Ying et al., 2011). The differentiation induced by retinoic acid produced chemosensitizing and radiosensitizing effects, and decreased the secretion of the angiocytokines VEGF and basic fibroblast growth factor (Campos et al., 2010). Apart from retinoic acid, the growing body of knowledge on the differentiation path of adult stem cells is fuelling alternative ways for forcing CSCs to differentiate. Bone morphogenetic proteins (BMPs), members of the TGF-β superfamily, instruct cell fate during neural development by interacting with their cognate receptors (BMPR), ultimately triggering the Smad signaling cascade. Based on this background, it has been demonstrated that BMP4 induces differentiation of GBM-SCs and inhibits tumor growth *in vitro* through the activation of the canonical signaling, a finding confirmed *in vivo* (Piccirillo et al., 2006). The connection between BMPs and stem cell differentiation has been further enforced by demonstrating that GBM-SCs can epigenetically regulate BMPRs, a process that produces a shift toward a fetal phenotype overcoming the pro-differentiation effects of BMPs. Consistently, the forced expression of BMPR1B restored the pro-differentiative effects of the pathway and impaired tumorigenicity (Lee et al., 2008). Recently, novel mechanistic insights on the role of BMP pathway have been provided. BMP2 was found to be able to sensitize GBM-SCs to temozolomide by inducing down-regulation of hypoxia-inducible factor-1α and MGMT (Persano et al., 2012). Investigators revealed that a BMP7 variant decreased GBM-SCs proliferation, endothelial cord formation, and stem cell marker expression *in vitro*, and reduced brain invasion, angiogenesis, and mortality in an orthotopic mouse model (Tate et al., 2012). Interferon-beta (IFN-β) has been proposed as a further differentiation-inducing agent. IFN-β-mediated differentiation led to reduced proliferation and self-renewal, producing chemosensitizing effects (Yuki et al., 2009). Finally, stem cell transcription factors including Sox2, Oct4, and Nanog are involved in the maintenance and functions of embryonic and adult stem cells (Cheng et al., 2010), and are critical for cell reprogramming and generation of inducible pluripotent stem cells (Takahashi and Yamanaka, 2006). Even though targeting these molecular effectors induces differentiation of GBM-SCs, it is still controversial whether or not these strategies can be translated to the clinical setting, being potentially burdened by ”off-target“ effects on tissue-resident stem cells.

## TARGETING THE HYPOXIA RESPONSE

Hypoxia is a hallmark of GBM. Low oxygen conditions are sensed by hypoxia inducible factors (HIFs), which mediate an adaptive response consisting in the production of angiogenic cytokines that stimulate the generation of new blood vessels. Newly formed vessels are, however, disorganized and dysfunctional, thus fuelling a vicious circle between hypoxia and neoangiogenesis. As discussed above, GBM-SCs directly participate in this aberrant loop through different biological mechanisms. It is known that there is a close connection between oxygen levels and stem cells (Cheng et al., 2010). Hypoxic niches are involved in maintaining normal stem cells, as demonstrated in the hematopoietic system. Furthermore, hypoxia prevents the differentiation of neural stem cells, promotes the self-renewal of embryonic stem cells, and enhances the generation of induced pluripotent stem cells. Similarly, it has been suggested that hypoxic niches are the environment in which CSCs thrive, while disrupting these privileged microenvironments may provide a new approach for targeting GBM-SCs. Functional studies through RNA interference revealed that both HIF1α and HIF2α are required for GBM-SCs (Li et al., 2009), and that hypoxia drives the expansion of GBM-SCs (Soeda et al., 2009). Interestingly, hypoxia differentially induces HIF members in stem versus non-stem GBM cells. While HIF1α is induced in both GBM-SCs and non-stem cells, HIF2α and its target genes are specifically up-regulated in GBM-SCs. Furthermore, HIF2α was not expressed in normal neural progenitors, thus suggesting that HIF2α inhibition might spare normal stem cells. Recently, it has been demonstrated that the autocrine axis VEGF-VEGF receptor 2 (VEGFR2)-neuropilin-1 drives GBM-SCs growth (Hamerlik et al., 2012). Authors found that the limited efficacy of bevacizumab was correlated with this type of autocrine signaling, which is associated with receptor recycling and the existence of a cytosolic pool of active VEGFR2. Conversely, GBM-SCs viability was affected by the inhibition of the VEGFR2 tyrosine kinase activity. Overall, studies mentioned above suggest that targeting hypoxia/neoangiogenesis may provide novel strategies for GBM-SCs targeting. Nevertheless, the study of the CSC-microenvironment interactions is only at the beginning, and a more in-depth characterization is required for improving the therapeutic potential of these compounds in the clinical scenario. **Table 1** summarizes pharmacological strategies investigated for targeting GBM-SCs

**Table 1:** Pharmacological strategies for targeting GBM-SCs.

Strategy	Targets	Reference
Canonical pathway inhibitors	EGFR, TGF-β, c-MET, PI3K/Akt, STAT3	Eyler et al. (2008), Bleau et al. (2009), Sherry et al. (2009), Anido et al. (2010), Jin et al. (2011), Joo et al. (2012)
Self-renewal pathway inhibitors	Sonic Hedgehog, Notch	Bar et al. (2007), Fan et al. (2010)
Differentiation-inducing agents	Retinoic acid, BMPs, IFN-β	Piccirillo et al. (2006), Yuki et al. (2009), Ying et al. (2011), Tate et al. (2012)
CSC-microenvironment disrupting agents	HIFs, VEGF/VEGFR axis, Notch	Li et al. (2009), Zhu et al. (2011), Hamerlik et al. (2012)
DNA damage response	Chk1 and Chk2	Bao et al. (2006b), Ropolo et al. (2009)

## GLIOBLASTOMA STEM CELLS IN THE CLINICAL SETTING

The discovery of CSCs added a further level of complexity to the biology of tumors. In the past decade, efforts in dissecting their functional properties allowed the first wave of translational investigations. Aims of these studies were to explore the correlation existing between CSC-related parameters and clinical outcomes. Different predictors were investigated, such as expression of putative stem cell markers (Pallini et al., 2008), sphere-forming efficiency (Laks et al., 2009), stem cell-related signatures (Liu et al., 2007), and polymorphisms in stem cell genes (Gerger et al., 2011). By evaluating CD133 expression by immunohistochemistry in a series of 95 gliomas of various grade and histology, authors reported an inverse correlation between the proportion of CD133^+^ cells, and their topological organization in clusters, and progression-free survival (PFS) and OS (Zeppernick et al., 2008). In this study, the association of CD133 expression and shorter PFS and OS occurred independently from established clinical and pathological prognostic factors such as tumor grade, extent of resection, and patient age. This finding was later confirmed in an independent study. The *in vitro* generation of CSCs and the expression of CD133 correlated with long-term outcomes, while the co-expression of CD133 and Ki67 identified a fraction of patients with very short PFS and OS (Pallini et al., 2008). The prognostic value of neurosphere formation and tumorigenic capacity was further confirmed in an analysis of 32 GBM patients (Laks et al., 2009). Notably, in multivariate analysis neurosphere generation remained a significant predictor of poor clinical outcomes. Increased CD133 expression was detected in recurrent GBM (Pallini et al., 2011). Surprisingly, CD133 expression was significantly associated with longer survival after tumor recurrence given that non-tumor neural stem cells represented 20–60% of CD133^+^ cells in this setting. Therefore, authors concluded that the recruitment of neural stem/progenitor cells from surrounding brain may exert anti-tumorigenic effects. As mentioned above, many attempts have been made for identifying stem cell-associated gene expression profiles that correlate with clinical parameters. By profiling 80 GBM, authors identified a cluster of deregulated genes dominated by HOX components, and therefore reminiscent of a ”self-renewal“ signature, which predicted poor survival and treatment resistance in patients receiving concomitant chemo-radiotherapy (Murat et al., 2008). The HOX signature maintained its negative prognostic value in multivariate analysis adjusted for MGMT methylation status. An independent HOX/stem cell gene signature was also correlated with shorter survival in pediatric high-grade glioma patients (Gaspar et al., 2010). Although studies discussed above suggest a prognostic impact of CSC-related endpoints, some considerations need to be taken into account. These studies were conducted in retrospective series, and they did not consider an independent validation set. Therefore, prospective and adequately powered trials are required for thoroughly understanding the prognostic and predictive significance of CSC-associated parameters. Finally, the molecular taxonomy GBM should be taken into account when exploring the correlation between CSCs and clinical outcomes. Several genes identifying a given subtype are also differentially regulated between CD133^+^ and CD133^-^, thus suggesting that distinct CSCs maintain different molecular subtypes (Günther et al., 2008; Joo et al., 2008; Lottaz et al., 2010).

## CONCLUSION

The identification of GBM-SCs challenged principles of experimental and medical oncology, and prompted novel therapeutic approaches. Nevertheless, most of the molecules endowed with anti-CSC activity in the preclinical setting did not show formal proof of effectiveness in clinical trials, highlighting the need for a more in-depth understanding of biological principles governing CSC fate. In this scenario, disease segmentation emerging from recent data is expected to enable more focused investigations. To this end, a thorough examination of different GBM-SC types is required, considering that each one potentially displays a peculiar biological behavior and a distinct spectrum of sensitivity to pathway-focused inhibitors. Furthermore, the CSC concept is changing. The original view that only CSCs are tumorigenic has been questioned, as well as the existence of a rigid hierarchy. To sum up, it is increasingly evident that a better definition of GBM-SCs is needed, ideally within a defined disease entity. Answering these questions will help delineate the appropriate clinical context for exploring effective anti-CSC therapies and CSC-related prognostic and predictive biomarkers.

## Conflict of Interest Statement

The authors declare that the research was conducted in the absence of any commercial or financial relationships that could be construed as a potential conflict of interest.
